# Does Decreased SNX10 Serve as a Novel Risk Factor in Atrial Fibrillation of the Valvular Heart Disease? - A Case-Control Study

**DOI:** 10.21470/1678-9741-2019-0413

**Published:** 2021

**Authors:** Jianping Yao, Jian Hou, Linhua Lv, Chen Song, Mingxia Zhang, Zhongkai Wu

**Affiliations:** 1 Department of Cardiac Surgery, The First Affiliated Hospital of Sun Yat-sen University, Guangzhou, People's Republic of China.; 2 NHC Key Laboratory of Assisted Circulation, Sun Yat-sen University, Guangzhou, Guangdong Province, People's Republic of China.

**Keywords:** Atrial Fibrillation, Troponin T, Brain Natriuretic Peptide, C-Reactive Protein, Sorting Nexins, Immunohistochemistry, Risk Factors, Autopsy, Cardiac Surgical Procedures, Fibrosis

## Abstract

**Introduction:**

Atrial fibrillation (AF) is the most common sustained arrhythmia. Sorting nexin 10 (SNX10) has been reported to be an important regulator in embryonic development and human diseases, however, little is known about its role in cardiac disease. The aim of this study was to investigate the clinical significance of SNX10 expression in AF.

**Methods:**

Nineteen valvular heart disease patients with AF and nine valvular heart disease patients with sinus rhythm (SR) were enrolled. Atrial tissue samples from patients undergoing open heart surgery were examined. Atrial tissues of normal hearts were obtained from two cases’ autopsies. The SNX10 expression and its associations with the degree of fibrosis were analyzed by immunohistochemistry and Masson’s trichrome staining.

**Results:**

SNX10 expression was detected in the cytoplasm of cardiac cells in human myocardial tissue. The SNX10 expression level was higher in the SR group than in the AF group (*P*=0.023). SNX10 expression was negatively associated with the degree of fibrosis (*P*=0.017, Spearman rho=-0.447), the New York Heart Association degree (*P*=0.003, Spearman rho=-0.545), left atrial diameter (*P*=0.038, Spearman rho=-0.393), right atrial diameter (*P*=0.043, Spearman rho=-0.386), and the brain natriuretic peptide (BNP) level 24 hours after surgery (*P*=0.030, Spearman rho=-0.426), but not the BNP level before surgery and 72 hours after surgery. No statistical significance was observed between SNX10 and the level of troponin T and C-reactive protein.

**Conclusion:**

Decreased SNX10 might serve as a potential risk factor in AF of the valvular heart disease.

**Table:** 

Abbreviations, acronyms & symbols				
**AF**	**= Atrial fibrillation**		**NYHA**	**= New York Heart Association**
**BNP**	**= Brain natriuretic peptide**		**PFA**	**= Paraformaldehyde**
**CRP**	**= C-reactive protein**		**RA**	**= Right atrial**
**Cx**	**= Connexin**		**RV**	**= Right ventricular**
**ECG**	**= Electrocardiogram**		**SNX10**	**= Sorting nexin 10**
**EF**	**= Ejection fraction**		**SNXs**	**= Sorting nexins**
**IL-1β**	**= Interleukin 1β**		**SPSS**	**= Statistical Package for the Social Sciences**
**IVS**	**= Interventricular septum**		**SR**	**= Sinus rhythm**
**LA**	**= Left atrial**		**TNF-α**	**= Tumor necrosis factor alpha**
**LVESD**	**= Left ventricular end-systolic diameter**		**TnT**	**= Troponin T**
**LVID**	**= Left ventricular internal diastolic**		**VHD**	**= Valvular heart disease**
**LVPW**	**= Left ventricular posterior wall**			

## INTRODUCTION

Atrial fibrillation (AF) is the most common cardiac arrhythmia and the cause of considerable morbidity, mortality, and health-related expenditures^[1]^. One of the clinical risk factors for the development of AF is valvular heart disease, which can cause structural atrial changes, including dilatation and fibrosis^[2-5]^. Structural changes underlying the atrial substrate, including atrial fibrosis and atrial dilatation, greatly contribute to permanent AF. Atrial fibrosis can change a homogeneously activated syncytial atrium into a discontinuous and branching structure susceptible for multiple wavelet re-entry^[6]^. On the other hand, dilated atria will also help to sustain AF since larger atria can harbor more re-entrant wavelets at the same time^[7]^. Thus, identifying the crucial genes which were involved in atrial structural remodeling can facilitate the significant advances in the understanding of the mechanisms associated with AF.

Sorting nexins (SNXs) are a family of evolutionarily conserved proteins containing a phox-homology domain, by which the SNXs can target to endosome membrane through binding with phosphoinositide to regulate endosomal cargo sorting and trafficking^[8]^. Some studies of SNXs in relation to cardiac diseases have been reported. Choi et al.^[9]^ found that SNX13 mediates the heart failure process by the degradative sorting of apoptosis repressor with caspase recruitment domain. Zhao et al.^[10]^ demonstrated that SNX17 is involved in acute myocardial infarction-related ventricular arrhythmias. Then, Chen Y et al.^[11]^ found that SNX17 deficiency leads to the cardiac K^+^ channel Kv1.5 retention on the plasma membrane, thus increasing the risk of AF onset. SNX10 knockdown in zebrafish results in heart looping randomized, suggesting its potential role in heart disease. However, little has been reported about the role of SNX10 in cardiac disease.

In the present study, we investigated the relationships between the SNX10 expression and AF as well as the fibrosis degree in valvular heart disease. Moreover, the relationships between SNX10 expression and the levels of troponin T (TnT), C-reactive protein (CRP), and brain natriuretic peptide (BNP) were also investigated.

## METHODS

### Ethics

This study was approved by the Human Ethics Committee of the First Affiliated Hospital of Sun Yat-sen University and complied with the principles governing the use of human tissues that are outlined in the Declaration of Helsinki. Informed consent was given before participation in the study.

### Patients and Control Subjects

The study involved two groups of subjects. Group 1 consisted of 19 valvular heart disease patients with AF. Group 2 consisted of nine valvular heart disease patients with sinus rhythm (SR). The diagnosis of AF was reached by evaluating medical records and 12-lead electrocardiogram (ECG) findings. SR patients had no history of using antiarrhythmic drugs and were screened to ensure that they had never experienced AF. Preoperative two-dimensional color transthoracic echocardiography was performed routinely on the patients. Preoperative functional status was recorded according to New York Heart Association (NYHA) classifications. All of these patients underwent valve replacement surgery. The right atrial (RA) appendage samples were obtained at the time of valve replacement surgery.

The two cases’ autopsies with normal hearts were also detected. The hearts obtained at autopsy were devoid of any abnormal findings and the causes of death were not heart-related.

### Human Tissue Preparation

Tissue samples from the RA appendage were obtained from 28 patients with valvular heart disease. All of the patients underwent valve replacement surgery. The abovementioned tissue samples were obtained at the time of valve replacement surgery and were immediately fixed in 4% paraformaldehyde (PFA). A diagnosis of AF was made based on patient medical records and 12-lead ECG findings. Preoperative functional statuses were recorded in accordance with NYHA classification. The patients’ data are summarized in Table 1.

**Table 1:** Patients' characteristics.

	AF (n=19)	SR (n=9)	*P*-value
Female/male	13/6	3/6	0.114
NYHA			
I+II	9	7	0.133
III+IV	10	2	
LA diameter	63.368±21.843	42.444±8.368	0.001
LVID diameter	52.737±10.402	53.000±7.106	0.938
LVESD	34.053±5.691	34.889±6.254	0.738
IVS	9.579±1.610	10.611±2.447	0.193
LVPW thickness	9.474±1.439	9.889±1.691	0.535
RA diameter	63.368±12.321	47.333±9.552	0.001
RV diameter	26.105±9.678	24.889±10.517	0.774
EF	62.526±7.597	62.667±7.000	0.962
TnT (24 hours after surgery)	1.550±1.437	1.104±0.674	0.387
TnT (48 hours after surgery)	1.231±0.878	0.924±0.768	0.363
CRP (24 hours after surgery)	17.101±28.845	17.230±25.823	0.991
CRP (48 hours after surgery)	77.111±43.832	97.237±40.029	0.273
BNP (before surgery)	1408.395±1695.566	887.325±735.775	0.415
BNP (24 hours after surgery)	2901.521±3279.847	1838.157±1236.003	0.242
BNP (72 hours after surgery)	5385.279±7469.039	6801.878±10401.187	0.722

P-value comparison between the two groups with Student's t-test or χ^2^-tests A P-value < 0.05 was considered statistically significant AF=atrial fibrillation; BNP=brain natriuretic peptide; CRP=C-reactive protein; EF=ejection fraction; IVS=interventricular septum; LA=left atrial; LVESD=left ventricular end-systolic diameter; LVID=left ventricular internal diastolic; LVPW=left ventricular posterior wall; NYHA=New York Heart Association; RA=right atrial; RV=right ventricular; SR=sinus rhythm; TnT=troponin T

The RA appendages of normal hearts were obtained from autopsies (two cases) and provided by the Department of Forensic Pathology of Shantou University Medical College and the Department of Forensic Pathology of Jiaxing University Medical College, which were reported previously^[12]^. Both patients were male, and they were 19 and 23 years old. The hearts obtained at autopsy were devoid of any abnormal findings and the causes of death were not heart-related.

### Immunohistochemical Staining

All the samples were fixed in 4% PFA, embedded in paraffin, and stained with hematoxylin and eosin for routine histological examination. Immunohistochemical staining was performed on 4-µm-thick tissue sections. After deparaffinization and rehydration, all the sections were microwaved (10 min) in 0.01 mol/L sodium citrate buffer (pH 6.0) for antigen retrieval. To block endogenous peroxidase activity, we incubated the sections with 10% normal goat serum in phosphate-buffered saline for 15 min at room temperature. Then, all the sections were incubated with a rabbit polyclonal antibody against SNX10 (1:100; Abcam, Cambridge, United Kingdom) overnight at 4 °C. The slides were subsequently treated with the SuperPic Ture Polymer Detection Kit and the Liquid DAB Substrate Kit (Zymed/Invitrogen, San Francisco, United States of America) and counterstained with hematoxylin, dehydrated, and mounted.

### Masson’s Trichrome Staining

The sections were stained with Masson’s trichrome for fibrosis quantification. For Masson’s trichrome staining, the slices were dewaxed with xylol (two dewaxing steps lasting 2 min each, followed by soaking in a series of graded alcohols with concentrations ranging from 95% to 99%). Then, all the slices were washed in distilled water and placed in a hematoxylin solution for 3 min, after which a color change was induced with lithium carbonate. The slices were subsequently washed in pure water and stained with Ponceau red staining (in an oven at 30 °C and at 45kW for 20 sec). Then, the slices were placed in acidic water and phosphomolybdic acid for 1 min before being labeled with a green fluorescent marker and washed with acidic water. Subsequently, fibrosis severity was assessed in each of the sections upon their collection.

### Immunostaining Evaluation

Immunohistochemical expression was evaluated using the Image-Pro Plus 6.0 software. Briefly, at least three fields with positive expression from one section of myocardial tissue were randomly selected, and then these positive regions were analyzed with Image-Pro Plus 6.0 to determine the integral optical density and area. The average optical density, which represented the expression intensity in the section, was subsequently calculated. The average of the optical density values was determined to represent the expression intensity in the section.

Fibrosis Evaluation

Fibrosis severity was evaluated using the Image-Pro Plus 6.0 software. At least three fields from one section of myocardial tissue were randomly selected after which the ratio of the fibrotic area to the total area of each selected field was calculated to assess fibrosis severity. The average ratio, which represented the severity of the fibrosis in the section of myocardial tissue, was subsequently determined.

### Statistical Analyses

Continuous variables are presented as the mean ± standard error of mean. Comparisons of continuous variables between groups were performed with Student’s *t*-test or χ^2^-tests, and the correlations between SNX10 expression levels and fibrosis severity as well as other clinical variables of patients were assessed with the non-parametric Spearman rank correlation test. A *P*-value < 0.05 was considered statistically significant. All statistical analyses were performed with the Statistical Package for the Social Sciences (SPSS) software, version 13.0 (SPSS Inc., Chicago, Illinois, United States of America).

## RESULTS

Table 1 shows the demographic data of the patients enrolled in this study. Patients with valvular heart disease consisted of 12 men and 16 women (age: 51.63±11.32 years). We retrieved information, including sex, age, NYHA degree, cardiac rhythm, values of echocardiography, BNP, TnT, and CRP levels, from the patients’ hospital charts.

**Fig. 1:**
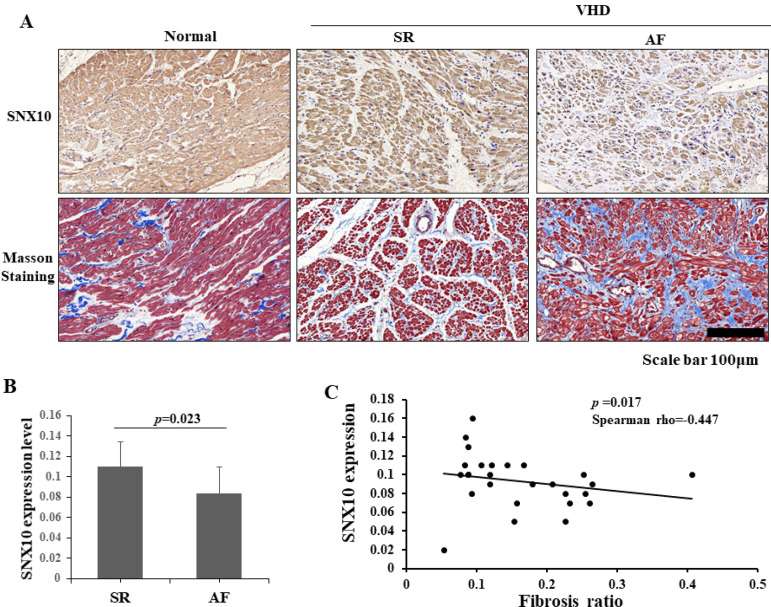
Histologic and immunohistochemical analysis of the myocardial tissues of VHD patients. (A) The SNX10 staining in the right atrial appendage of normal heart and the SR and AF samples of VHD patients. Masson’s staining for fibrosis severity was also detected. (B) The expression level of SNX10 in the AF and SR samples. (C) The association between the SNX10 level and the fibrosis degree in VHD patients. AF=atrial fibrillation; SNX10=sorting nexin 10; SR=sinus rhythm; VHD=valvular heart disease. Scale bars: 100 µm.

SNX10 expression was detected in the cytoplasm of cardiac cells in human myocardial tissue (Figure 1A). Strong staining for SNX10 was detected in the normal human tissue, and lower expression was observed in the myocardial tissue from the patients with valvular heart disease (Figure 1A). Moreover, the expression level of SNX10 was higher in the SR group than in the AF group (Figure 1B, *P*=0.023), and was negatively associated with the degree of fibrosis (Figure 1C, *P*=0.017, Spearman rho=-0.447, a moderate correlation), suggesting that decreased SNX10 is involved in AF. Furthermore, SNX10 expression was negatively associated with NYHA degree (*P*=0.003, Spearman rho=-0.545, a moderate correlation), left atrial diameter (LA) (*P*=0.038, Spearman rho=-0.393, a modest correlation), RA diameter (*P*=0.043, Spearman rho=-0.386, a modest correlation), and the BNP level 24 hours after surgery (*P*=0.030, Spearman rho=-0.426, a moderate correlation), but not the BNP level before surgery and 72 hours after surgery (Table 2). No statistical significance was observed between SNX10 and the level of TnT and CRP (Table 2). Taken together, these results suggest that decreased SNX10 might serve as a risk factor in AF of the valvular heart disease.

**Table 2:** The associations between the SNX10 expression level and the clinical parameter of patients with valvular heart disease.

	SNX10 expression level	
	Spearman rho	*P*-value
NYHA degree	-0.545	0.003
LA diameter	-0.393	0.038
RA diameter	-0.386	0.043
LVID diameter	0.087	0.661
LVESD	0.045	0.819
IVS	0.087	0.661
LVPW thickness	0.221	0.259
EF	0.122	0.538
TnT (24 hours after surgery)	-0.034	0.862
TnT (48 hours after surgery)	-0.157	0.434
CRP (24 hours after surgery)	-0.075	0.715
CRP (48 hours after surgery)	0.215	0.301
BNP (before surgery)	-0.166	0.408
BNP (24 hours after surgery)	-0.426	0.030
BNP (72 hours after surgery)	0.065	0.746

The correlations between SNX10 expression levels and the patients' clinical variables above were assessed with the non-parametric Spearman rank correlation test A P-value < 0.05 was considered statistically significant BNP=brain natriuretic peptide; CRP=C-reactive protein; EF=ejection fraction; IVS=interventricular septum; LA=left atrial; LVESD=Left ventricular end-systolic diameter; LVID=left ventricular internal diastolic; LVPW=Left ventricular posterior wall; NYHA=New York Heart Association; RA=right atrial; SNX10=sorting nexin 10; TnT=troponin T

## DISCUSSION

SNX10 has been found to play an important role in embryonic development^[11]^, alcohol-induced liver injury and steatosis^[13]^, osteoclast formation and resorption activity^[14,15]^, colorectal cancer^[16]^, and phagosome maturation in macrophages^[17]^. However, little is known about its role in cardiac disease. In the present study, we first found that strong SNX10 staining was detected in the normal human tissue, suggesting its potential important role in cardiac function. Moreover, we firstly reported the association between SNX10 and the valvular heart disease. Decreased SNX10 expression was related with AF and higher levels of the fibrosis degree, NYHA degree, LA diameter, and RA diameter, suggesting the important role of SNX10 in cardiac disease.

AF, the most common sustained arrhythmia, confers an independent increased risk of death^[2,18]^. The process of AF involves a structural remodeling, of which connective tissue deposition and fibrosis are the hallmarks, as well as altered atrial electrophysiological properties that facilitate the initiation and perpetuation of AF^[19]^. In our study, SNX10 expression was significantly negatively associated with the level of the fibrosis degree, LA diameter, and RA diameter, suggesting that SNX10 may be involved in AF by affecting cardiac remodeling in the valvular heart disease. Furthermore, given that BNP was proposed to enable development of novel tools to improve clinical risk assessment in AF^[20]^, the relationship between SNX10 and BNP was also investigated in our study. However, we did not observe a statistically significant difference in the BNP levels between the AF and SR groups (Table 1), which may have been because of an insufficient sample size. Interestingly, SNX10 expression was associated with BNP level 24 hours after surgery (Table 2), and the use of BNP for the diagnosis and management of heart failure is well established^[21,22]^. Thus, our data suggests that SNX10 might be a potential prognosis marker for the valvular heart disease.

The regulatory mechanism of SNX10 in cardiac disease remained unknown. SNX10 has been reported to regulate endosomal morphology, which might be crucial for macrophage function^[17]^. Activated macrophages cause AF mainly through tumor necrosis factor alpha (TNF-α) and interleukin 1β (IL-1β). TNF-α causes downregulation of connexin (Cx) 40 and Cx 43, atrial fibrosis, altered Ca^2+^ handling, and increased cardiocyte apoptosis and myolysis, while IL-1β inhibits protein quaking 1 expression in atrial myocyte and results in L-type Ca^2+^ current downregulation^[23]^. However, SNX10 knockout in mice reduces the serum levels of TNF-α and IL-1β, resulting in the suppression of immune inflammation and bone erosion in rheumatoid arthritis. It has been proposed that the complexity of SNX10 function may be species-specific and organ-specific. However, more investigations need to be done.

## CONCLUSION

In summary, our study is the first to report an association between SNX10 and the valvular heart disease. Decreased SNX10 expression was related to AF and higher levels of fibrosis degree, NYHA degree, LA diameter, and RA diameter, suggesting the important role of SNX10 in cardiac disease. SNX10 was negatively associated with BNP level 24 hours after surgery, suggesting its potential value as a prognostic marker for the valvular heart disease.

**Table:** 

Authors' roles & responsibilities	
JY	Drafting the work; final approval of the version to be published
JH	Analysis of data for the work; drafting the work; final approval of the version to be published
LL	Acquisition of data for the work; final approval of the version to be published
CS	Acquisition of data for the work; final approval of the version to be published
MZ	Acquisition of data for the work; final approval of the version to be published
ZW	Supervision; final approval of the version to be published
